# Synthesis and characterization of Ni_0.5_Al_0.5_Fe_2_O_4_ nanoparticles for potent antifungal activity against dry rot of ginger (*Fusarium oxysporum*)

**DOI:** 10.1038/s41598-022-22620-3

**Published:** 2022-11-22

**Authors:** Sushma Sharma, Poonam Kumari, Priyanka Thakur, Gaganpreet Singh Brar, Nahla A. Bouqellah, Abd El-Latif Hesham

**Affiliations:** 1 Dr Khem Singh Gill Akal College of Agriculture, Eternal University, H.P., Baru Sahib, Sirmour, India; 2 Akal College of Basic Science, Eternal University, H.P., Baru Sahib, Sirmour, India; 3 Dr Yashwant, Singh Parmar University of Horticulture and Forestry, H.P., Nauni, Solan, India; 4 Science College, Biology Department, Taibah University, Al-Madinah Al-Munawarh, 42317-8599 Saudi Arabia; 5 Department of Genetics, Faculty of Agriculture, Beni-Suef University, 62521, Beni-Suef, Egypt

**Keywords:** Microbiology, Nanoscience and technology

## Abstract

Current study signifies the use of nanoparticles as alternative in plant disease management to avoid harmful effect of pesticide and fungicide residue. Synthesis of nanoparticles (Ni_0.5_Al_0.5_Fe_2_O_4_) by hydrothermal method and studied their X-ray diffraction analysis (XRD), Raman spectra, and UV spectra and further successfully evaluated for antifungal activity against a soil and seed borne pathogenic fungus (*Fusarium oxysporum*).Among various pests, fungal pathogens are the main cause of crop destruction and we developed nanoparticles (Ni_0.5_Al_0.5_Fe_2_O_4_) which is successfully evaluated for antimycotic activity against dry rot (*F. oxysporum*) of ginger which causes 50–70% losses in the ginger plant. In vitro and in vivo analysis designated that the nanoparticles (Ni_0.5_Al_0.5_Fe_2_O_4_) has shown an excellent antifungal activity against *F. oxysporum* at 0.5 mg/ml concentration. Similarly, no disease incidence was recorded when Ni_0.5_Al_0.5_Fe_2_O_4_ nanoparticles used at 0.5 mg/ml concentration under in vivo conditions. In plants various environmental stresses (biotic and abiotic) leads to excessive production of reactive oxygen species (ROS) causing progressive oxidative damage and ultimately leads to cell death. The role of ROS in nanoparticles (Ni_0.5_Al_0.5_Fe_2_O_4_) represents by reduction in the growth inhibition of *F. oxysporum*. We speculated in light of these results that the cytotoxic effect of Ni_0.5_Al_0.5_Fe_2_O_4_ nanoparticles on *F. oxysporum* may be mediated through ROS. We can suggest the role of nanoparticles (Ni_0.5_Al_0.5_Fe_2_O_4_) gives a promising result as a fungicidal activity and could be a novel family of future new generation fungicide.

## Introduction

Currently agriculture is facing a number of problems, such as climate change, water pollution, soil pollution from different damaging environmental contaminants such as fertilizers and pesticides, to feed a huge population due to increasing food demands to feed worldwide population. Plant pests and diseases have caused large number of human losses to agriculture crops from the beginning of agriculture^1,2^. Among various pests fungi, bacteria, protozoa, viruses, and plant parasites are various infectious organisms that causes severe losses and serious infectious disease in plants. Plant pathogenic fungi are the most deadly pathogens among all of these pathogens, causing the most serious plant disorders. Fungal pathogens are considered to be most responsible for around causing 85% losses of all plant diseases^3^. To control these pathogens farmers are mostly dependent on using a variety of chemical fungicides such as mancozeb, copper hydroxide, kitazin and others, to handle different infections^4^. As a result, with the increased demand to manage infections, particularly fungus, it is important to address the overuse and indiscriminate use of fungicides by developing ecofriendly alternatives. Nanoparticle (NP) materials have gotten a lot of interest lately because of their distinct physical and chemical features that set them apart from their macroscale counterparts^5^. Various NP materials have been shown to have antibacterial and antifungal properties, such as silver^6^ Au-titanium dioxide nanowires^7^ zinc oxide^8^ copper^9^ CeO_2_ nanoparticle^10^, magnesium oxide^11^. Nanoparticles as pesticides or fungicides will only be advantageous or helpful in practical applications whether they are selective in eliminating infections while causing minimum or no damage to the environment. The present study successfully signifies the antifungal effect of nanoparticles against this important plant pathogenic fungus, *F. oxysporum*. Pathogen is very destructive in nature and resulting in huge losses to economically important agricultural crops. One or more different *Fusarium* species can infect many important crop plants grown on the earth. Pathogen mainly present in soil, seed and causes vascular wilt, dry rot, root rot in a variety of plants species^12^. Nano-fungicides, nano-pesticides, and nano-herbicides, for example, are being researched for use in agricultural activities^13–15^. Nanomaterials as nanofertilizers and nanopesticides^16,17^. The effect of nanoparticles varies depending on the plant species and kind of nanoparticle used. Numerous research have been conducted to demonstrate the beneficial effects of metal oxide and metal nanoparticle exposure on disease reduction and crop productivity. Zarinkoob et al. reported Ce-Mn ferrite nanocomposite promoted the photosynthesis, fortification of total yield, and elongation of wheat (*Triticum aestivum* L.)^17^. Silver, zinc oxide, magnesium, silicon, and TiO_2_ nanoparticles, for example, have been found to be engaged in the direct suppression of crop due to their antibacterial and antifungal properties^18^. For example, the antifungal effects of Fe-doped ceria nanoparticles for cytotoxic and antifungal activity^19^, Gum-based cerium oxide nanoparticles for antimicrobial assay^20^ and silver nanoparticles and ions on *Magnaporthe grisea* and *Bipolaris sorokiniana* were also investigated^21,22^. They found a decrease in the progression of disease by phytopathogenic fungi after treatment with both silver nanoparticles and silver ions in an in vitro and in vivo investigation. In field studies^23^,the silver nanoparticles suppressed the *Colletotrichum* spp. (anthracnose disease). Fungal mycelial growth is effectively inhibited by ferrite nanoparticles, according to our findings^24^. It was also shown that zinc oxide nanoparticles in Mung bean inhibited *Fusarium graminearum* growth when compared to bulk oxide and a control^25^. Quantum dots have also been shown to increase plant development in a number of studies, most likely due to their selective activity against pathogens^26^. Currently, they have confirmed that the pathogen *F. oxysporum* internalised quantum dots (500 nM) within the cells and detected a 20% reduction in fungus growth and a 15% reduction in hyphae development^26^. Suitable strategies adopted by plants in presence of NMs under challenging environments are also reported by Hassanisaadi^27^. Biosynthesis encloses the natural reduction property catalyzed by biomolecules in plants and microorganisms to transform metal ions into metal NPs. Hydrothermal synthesis offers many advantages such as relatively mild operating conditions (reaction temperatures < 300 °C), one-step synthetic procedure, environmental friendliness and good dispersion in solution. Nanomaterials with high vapour pressures can be produced by the hydrothermal method with minimum loss of materials. Hydrothermal synthesis used to obtaining small-sized fine particles^28,29^. Furthermore, the activity of Ni_0.5_Al_0.5_Fe_2_O_4_ nanoparticles in plants successfully investigated under in vivo conditions. Present study signifies percent mycelial effects of nanoparticles at different concentration were observed against *Fusarium* dry rot in ginger plants, which causes serious losses in storage and field conditions.

## Methods

### Synthesis of NAFO nanoparticles

To prepare Ni_0.5_Al_0.5_Fe_2_O_4_ nanoparticles, nickel nitrate, nonahydrate iron (III) nitrate, nonahydrate aluminum nitrate, and cetyltrimethy lammonium bromide (CTAB) and NaOH are used as precursors. In brief, Ni(NO_3_)_2_·6H_2_O (Sigma Aldrich), Al(NO_3_)_3_. 9H_2_O (Sigma Aldrich) and Fe(NO_3_)_3_·9H_2_O (Sigma Aldrich)were taken and dissolved in 60 mL of deionized water (DIW)to dissolve the precursors in stoichiometric proportions using magnetic stirrer. Drop wise addition of NaOH (g) to the solution (in ml) in a ratio of 4:1 (nitrates: NaOH) until the pH value reaches 11. The mixture is then vigorously agitated for 3 h afore being placed to a Teflon-lined steel autoclave (300 mL). In a muffle furnace, the sealed autoclave was heated to 130 °C for 48 h. Teflon-lined steel autoclave was slowly cool down to room temperature after 48 h. The autoclave precursors are filter out and then washed with acetone and D.I.W. Many times until the pH value is reduced to neutral or 7. The acquired slurry was dried overnight at 60 °C in oven, then dried powder calcined for 5 h at 500 °C in furnace. X-ray powder diffractometer (XRD) (PanlyticalX’pert Pro), Raman spectroscopy, SEM (JSM 6100 (JEOL)) Fourier transform infrared spectroscopy (FTIR; Cary 630 FTIR, Agilent Tech.) and UV–vis spectroscopy (Evolution 220 UV–Vis Spectrophotometer, Thermo Scientific™) were used to characterize the sample, which was then examined for antimycotic activity against plant-pathogenic fungus.

**Figure 1:**
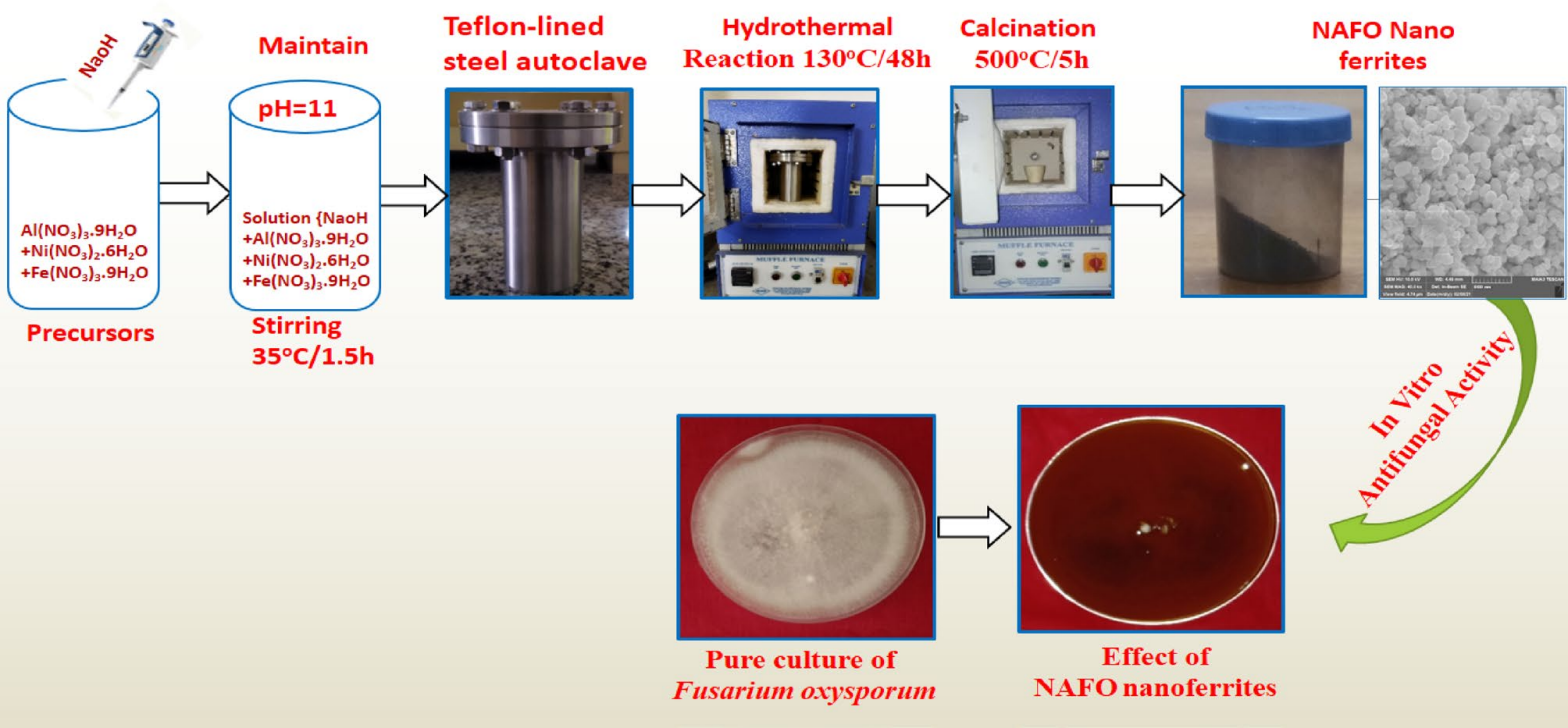
Schematic representation of NAFO nanoparticles preparation and its antifungal activity.

Better homogeneity, high purity, low processing temperature, ultra-fine size, and uniform phase distribution in multi-component systems like ferrite are some of the salient advantages of the hydrothermal synthesis approach^30,31^. Final black powder was obtained and whole preparation process is shown in Fig. 1.

### Isolation of pathogen

Isolation of the pathogen carried out from the stored infected ginger samples and then placed in potato dextrose agar (PDA) medium under aseptic conditions. Small bits of about 2–3 mm from infected ginger were cut using a surgical sterilized blade and then washed thoroughly with sterile distilled water and then further dip in 0.1 per cent sodium hypochlorite solution. Small bits were further blotted dry from 5 min to avoid moisture in laminar air flow chamber in aseptic conditions and then further these bits were placed on PDA plates. Further, these plates incubated in BOD incubator at 27 ± 1 °C for obtain the optimum growth of the fungal mycelium. Pure culture from a mixed fungal culture is obtained by using a single spore or a bit of fungal mycelium inoculated on PDA agar medium or in slants. Pure culture were sub cultured regularly after every 10 days of interval of time^32,33^.

### Characterization (microscopic and molecular) of pathogen

After one week of incubation a loopful portion of white cottony growth of crushed mycelium were placed on a clean glass slide cover with coverslip were further observed for microscopic examination (Dewinter Optical, Inc., New Delhi, India). Various morphological characteristics of the isolated fungus such as mycelium structure, microconidia and macroconidia arrangement were examined. For molecular characterization, The universal primers ITS1-F (5ʹ-CTTGGTCATTTAGAGGAAGTAA-3ʹ) and ITS4-R (5ʹTCCTCCGCTTATTGATATGC-3ʹ)^34^ were used for amplification of the internal transcribed spacer (ITS) region of the pathogenic fungi. PCR product for the isolates were sequenced and aligned with known ITS sequences in GenBank database^35^ (Table 1).

**Table 1:** GenBank accession numbers of the Fusarium isolates.

Sr. No	Isolate	Place of collection	Best match	GenBank Acc	S.No	Isolate	Place of collection	Best match	GenBank Acc
1	FUO (A)	Rajgarh, Sirmaur 1	*Fusarium* sp.	OM311625.1	21	FUO (9)	Oachghat,Solan 1	*Fusarium graminearum*	KJ847741.1
2	FUO (B)	Rajgarh, Sirmaur 2	*Fusarium oxysporum*	OM311626.1	22	FUO (5)	Oachghat,Solan 2	*Fusarium sp.*	KJ847725.1
3	FUO (G)	Rajgarh, Sirmaur 3	*Fusarium oxysporum*	KJ847726.1	23	FUO (X)	Oachghat,Solan 3	*Fusarium sp.*	KJ847742.1
4	FUO (11)	Rajgarh, Sirmaur 4	*Fusarium oxysporum*	KJ847727.1	24	FUO (Y)	Oachghat,Solan 4	*Fusarium* sp.	KJ847743.1
5	FUO (10)	Rajgarh, Sirmaur 5	*Fusarium proliferatum*	KJ847729.1	25	FUO (41)	Oachghat,Solan 5	*Fusarium oxysporum*	KJ847744.1
6	FUO (f)	Nauradhar,Sirmaur 1	*Fusarium s*p.	KJ847730.1	26	FUO (42)	Solan Market 1	*Fusarium oxysporum*	KJ847745.1
7	FUO (3)	Nauradhar,Sirmaur 2	*Fusarium oxysporum*	OM311627.1	27	FUO (43)	Solan Market 2	*Fusarium oxysporum*	KJ847746.1
8	FUO (8)	Nauradhar,Sirmaur 3	*Fusarium oxysporum*	OM311628.1	28	FUO (44)	Solan Market 3	*Fusarium oxysporum*	KJ847747.1
9	FUO (M)	Nauradhar,Sirmaur 4	*Fusarium oxysporum*	OM333235.1	29	FUO (K)	Solan Market 4	*Fusarium oxysporum*	OM311629.1
10	FUO (1)	Nauradhar,Sirmaur 5	*Fusarium equiseti*	KJ847732.1	30	FUO (52)	Solan Market 5	*Fusarium oxysporum*	KJ847749.1
11	FUO (2)	Bharadi, Bilaspur 1	*Fusarium oxysporum*	KJ847733.1	31	FUO (55)	Sabji Mandi, Shimla 1	*Fusarium oxysporum*	KJ847750.1
12	FUO (18)	Bharadi, Bilaspur 2	*Fusarium sp.*	OM333236.1	32	FUO (62)	SabjiMandi,Shimla 2	*Fusarium graminearum*	OM311630.1
13	FUO (14)	Bharadi, Bilaspur 3	*Fusarium graminearum*	KJ847734.1	33	FUO (63)	Sabji Mandi, Shimla 3	*Fusarium oxysporum*	KJ847752.1
14	FUO (25)	Bharadi, Bilaspur 4	*Fusarium verticillioides*	KJ847735.1	34	FUO (U)	Sabji Mandi, Shimla 4	*Fusarium* sp.	KJ847753.1
15	FUO (15)	Bharadi, Bilaspur 5	*Fusarium oxysporum*	KJ847736.1	35	FUO (UN)	Sabji Mandi,Shimla 5	*Fusarium* sp.	KJ847754.1
16	FUO (17)	Nauni campus, Solan 1	*Fusarium oxysporum*	KJ847737.1					
17	FUO (J)	Nauni campus, Solan 2	*Fusarium oxysporum*	KJ847724.1					
18	FUO (Q)	Nauni campus, Solan 3	*Fusarium oxysporum*	KJ847739.1					
19	FUO (T)	Nauni campus, Solan 4	*Fusarium solani*	KJ847740.1					
20	FUO (L)	Nauni campus, Solan 5	*Fusarium redolens*	OM333238.1					

### In vitro antifungal activity of (Ni_0.5_Al_0.5_Fe_2_O_4_) nanoparticles

**Figure 2:**
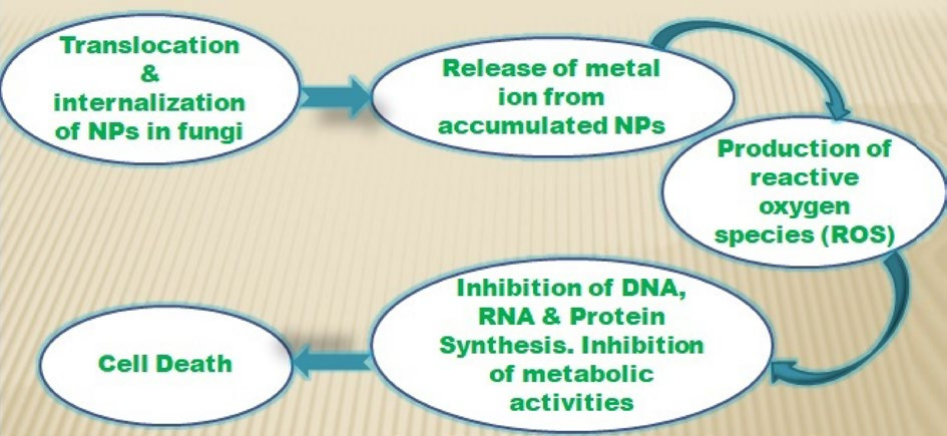
Flow chart representing mode of mechanism of NAFO nanoparticles against fungi.

To know the effectiveness of nanoparticles (Ni_0.5_Al_0.5_Fe_2_O_4_) against phytopathogenic fungus, *F. oxysporum*, nanoparticles (Ni_0.5_Al_0.5_Fe_2_O_4_) was tested firstly under laboratory conditions. The (Ni_0.5_Al_0.5_Fe_2_O_4_) nanoparticles were evaluated in vitro conditions and each treatment replicated five times to avoid the chances of error (Poisoned Food Technique)^36^. Different doses of nanoparticles (0.1 mg/ml, 0.2 mg/ml, 0.3 mg/ml, 0.4 mg/ml and 0.5 mg/ml) prepared and evaluated against plant pathogenic fungi. In in vitro evaluation double strength potato dextrose agar medium was prepared in distilled water and steam sterilized in an autoclave for 20 min, further different concentrations of nanoparticles prepared in sterile distilled water and sonicated for 30 min to produce a colloidal solution of nanoparticles. To produce a colloidal solution of (Ni_0.5_Al_0.5_Fe_2_O_4_) nanoparticles twofold concentrations were prepared and then further sonicated for 30 min. Prepared solution was further combined aseptically in laminar air flow with double strength medium and poured into petri plates and further this mixture is allow to solidify for one day to avoid moisture contamination. Next day, with the help of cork borer these plates were further inoculated with 3 mm bit of mycelial of 6 days old culture of the fungus. There was also a separate control treatment in which there was only sterilized distilled water. The inoculated plates were further incubated at 27 °C in incubator until the control plates fully covered with mycelium. The results reported as radial growth of plant-pathogenic fungus in millimeters (mm). The mycelial growth (per cent inhibition) of the pathogen was calculated^37^.

1 $$I = \frac{C - T}{C} \times 100$$

where, I-mycelial growth inhibition (%), C- mycelial (linear) growth in control (mm) after 24 h, T- mycelial growth (linear) in treatment (mm). Flow chart given in Fig. 2 representing mechanisms of NAFO nanoparticles actions against fungi**.**

### Determination of minimal fungicidal concentration (MFC) of nanoparticles Ni_0.5_Al_0.5_Fe_2_O_4_

Using different concentrations of nanoparticles (Ni_0.5_Al_0.5_Fe_2_O_4_) antifungal tests were performed by measuring the growth curve of *F. oxysporum* incubated in NB + 4% glucose broth medium. All of the trials were carried out in complete darkness. Specific amounts of nanoparticles (Ni_0.5_Al_0.5_Fe_2_O_4_) were fed to *F. oxysporum* culture (5 ml) with an initial OD of 0.1 at 600 nm in a typical experiment for the development of the growth curves. Cultures were cultivated with agitation at 36.5 °C (200 rpm). The optical density (OD_600_) of the sample was measured over time to estimate the growth curve. As a control, a blank containing NB + 4% glucose broth medium was cultured under the same circumstances. Experiments were carried out three times with each concentration. To see if nanoparticles (Ni_0.5_Al_0.5_Fe_2_O_4_) could stop *F. oxysporum* from growing in the exponential phase, the cells were grown in NB + 4% glucose until they reached an OD_600_ of 0.4–0.5 (log phase), then centrifuged and resuspended in fresh medium to an OD_600_ of 0.1, and nanoparticles were added at the indicated concentrations. To create the growth curve, the optical density (OD_600_) of the sample was measured over time.

### Histidine inhibits the (***F. oxysporum***) antifungal effect caused by (Ni_0.5_Al_0.5_Fe_2_O_4_) nanoparticles

Previous studies reported that different NPs are having capacity of producing ROS (Reactive oxygen species) in water suspensions. Present experiment is conducted to study the effect of histidine on the growth curve of *F. oxysporum* with nanoparticles Ni_0.5_Al_0.5_Fe_2_O_4_. Cultures were grown at 36.5 °C with agitation (200 rpm). The growth curve was determined by measuring the time evolution of the OD_600_ of the sample. *F. oxysporum* in NB + 4% glucose broth medium was used as a control. In addition, *F. oxysporum* with nanoparticles Ni_0.5_Al_0.5_Fe_2_O_4_cultured under the same conditions was used as a control to look at the effect of histidine on *F. oxysporum.* The inhibitory effect of histidine on nanoparticles Ni_0.5_Al_0.5_Fe_2_O_4_toxicity was tested by adding histidine to *F. oxysporum* cultures containing nanoparticles Ni_0.5_Al_0.5_Fe_2_O_4_at 0.05 mg ml^-1^ concentration. By introducing histidine (1 mM, 2 mM, 2.5 mM) to *F. oxysporum* cultures containing nanoparticles Ni_0.5_Al_0.5_Fe_2_O_4_at a dosage of 400 and 500 ppm, the inhibitory effect of histidine on nanoparticles Ni_0.5_Al_0.5_Fe_2_O_4_ toxicity was investigated.

### In vivo evaluation of antifungal activity of (Ni_0.5_Al_0.5_Fe_2_O_4_) nanoparticles

An experiment is conducted in polyhouse in pots to know the efficacy of (Ni_0.5_Al_0.5_Fe_2_O_4_) nanoparticles against dry rot of ginger (*F. oxysporum*). Method of preparation of inoculum: In pot experiment, rhizomes of variety "Suprabha" which is susceptible to dry rot disease in each pot were treated with different concentration of (Ni_0.5_Al_0.5_Fe_2_O_4_) nanoparticles (0.1 mg/ml, 0.2 mg/ml, 0.3 mg/ml, 0.4 mg/ml and 0.5 mg/ml) and sown in (@ 2 rhizomes/pot) disinfected with *F. oxysporum* sick soil. Rhizomes of ginger "Suprabha" variety dipped for 45 min in a solution containing varying concentrations of (Ni_0.5_Al_0.5_Fe_2_O_4_) nanoparticles and then rhizomes planted in pots and each treatment replicated 4 times with an adequate control. Before sowing (10 days before) inoculum of the mass culture of *F. oxysporum* prepared on sand maize medium (1:1) medium was added to the soil in pot @ 200gm/kg of soil. After transplantation pots were kept in polyhouse with a relative humidity of 60–70 percent. Pots were watered regularly and disease incidence was calculated until symptoms occurred in the control treatment.

2 $$Disease \;incidence \left( \% \right) = \frac{Number \;of \;plants \;infected}{{Number\; of\; plants \;observed}} \times 100$$

### Ethical approval and consent to participate

This method was carried out in accordance with relevant guidelines of IUCN policy statement on research involving species at risk of extinction and the convention on the trade in endangered species of wild fauna and flora.

## Results and discussion

### Fungal isolation and molecular identification

Thirty-five fungal pathogens were isolated from the stored infected ginger samples collected from different locations of Himachal Pradesh, India. Morphological characteristics indicated that all fungal isolates were belonged to the genus of *Fusarium.* PCR study showed amplified ranged from 565 ~ 615 bp in the isolates and on BLAST, most of the isolates showing high homologies (99 ~ 100%) with *F. oxysporum*. Therefore, *F. oxysporum* (KJ847727.1) species was selected to study the effect of nanoparticles Ni_0.5_Al_0.5_Fe_2_O_4_ on their mycelial growth. The sequences further submitted to comprehensive database NCBI and its GenBank Accession Numbers for the isolates as shown in Table 1.

### XRD and Raman spectroscopy analysis of NAFO nanoparticles

**Figure 3:**
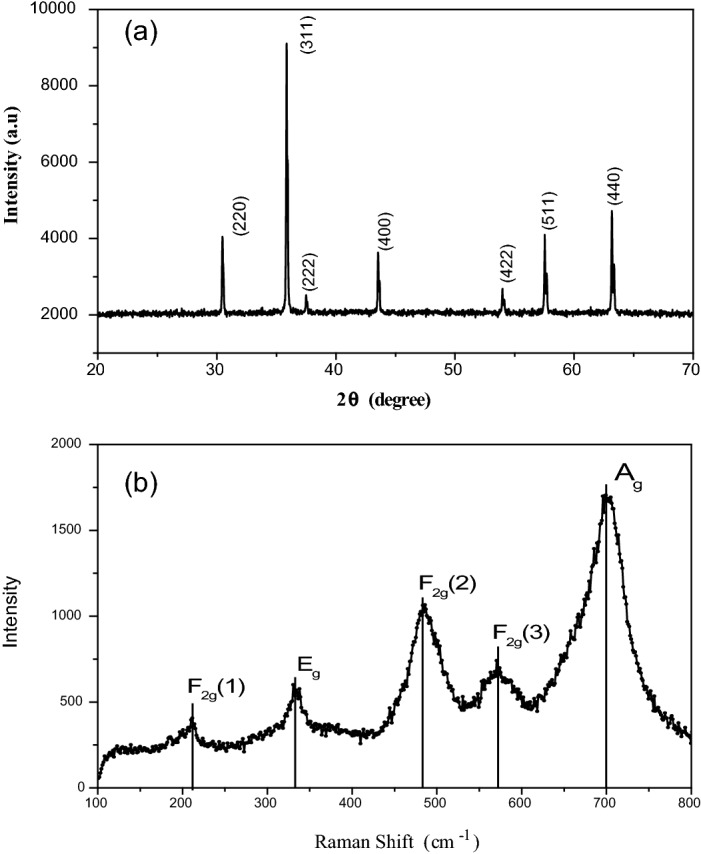
(**a**) XRD pattern of NAFO nanoparticles (**b**) Raman spectra of NAFO nanoparticles.

X-ray diffraction technique was used to examine phase formation of the nanoparticles at room temperature using Philips X-ray diffractometer (PW 3710) with Cu-Kα radiation. The 2θ range of diffraction patterns was recorded from 20 to 80°. The major lattice planes as given in Fig. 3 (a) confirms the construction of spinel structured cubic nickel ferrite according to Card No. 10–0325 through no additional phases or impurities existent. The Fd3m space group represented in this single-phase cubic spinel structure. The reflection line (311) was used for obtaining the crystallite size using the Scherrer^38^ Eq. (3)

3 $${\text{D}} = \frac{{0.9\lambda }}{{\beta \;{\text{Cos}}\theta _{{\text{B}}} }}$$

where, D is the particle diameter, λ is 0.154 nm. The broadening of the XRD peaks is suggesting presence of the nano-sized crystals. We use Scherrer relationship (Eq. (3)) to estimate have an average crystalline size of 46 nm.

For confirmation of phase purity of NAFO nanoparticles, Raman spectra were also recorded at room temperature as presented in Fig. 3b. Figure 3b shows that the sample have active modes, as expected in the spinel structure by group theory^39^. The typical appearance of these modes is shown as:A_g_ + E_g_ + 3 F_2g_. The A_g_ mode corresponds to the symmetric stretching of Fe–O and Ni–O bonds at the tetrahedral group site. E_g_ mode corresponds to symmetric bending of Fe–O and Ni–O bonds at the tetrahedral site. The F_2g_(2) mode is assigned to the asymmetric stretching of Fe–O and Ni–O bonds at the octahedral site. F_2g_(3) mode is caused due to the asymmetric bending of Fe–O and Ni–O bonds at the octahedral site, and F_2g_(1) mode is due to translational motion of the whole tetrahedral group. Samples are given in the Table 2 with reference of all five active modes F_2g_ (1), E_g_, F_2g_ (2), F_2g_ (3) and A_g_. From Fig. 3b and Table 2, it is observed easily that some of the Raman signals are very broad, suggesting that they may be composed of more than one Raman band and there is shift of Raman peaks towards higher wave number. Raman band shifts to higher frequency and becomes narrow. This behavior is typical of confinement of phonons due to particle size reduction^39^.

**Table 2:** Parameters calculated from Raman spectra of NAFO nanoparticles by using intense peaks.

Modes (↓)	Sample ( →)	Raman shift (cm^−1^) ± 1
F_2g_ (1) (translational motion)	Tetrahedral group site	212
E_g_ (symmetric bending)	Tetrahedral group site	334
F_2g_ (2) (asymmetric stretching)	Octahedral group site	486
F_2g_ (3) (asymmetric bending)	Octahedral group site	571
A_g_ (symmetric stretching)	Tetrahedral group site	703

### SEM study of NAFO nanoparticles

**Figure 4:**
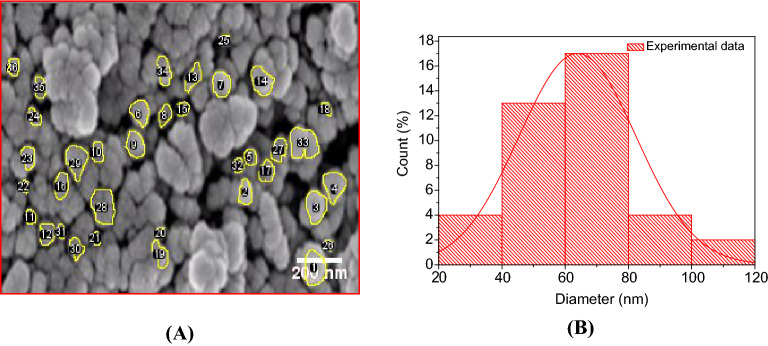
(**A**) SEM image and (**B**) Average particle size distribution (histogram) from SEM image by Image J software of NAFO nanoparticles.

Figure 4A shows SEM image of NPs reveal agglomeration and spherical shape nanoparticles with a small particle size. The intercept technique used to calculate the sample’s average grain size by ImageJ software. The SEM image with selected particles of the nanoparticles Ni_0.5_Al_0.5_Fe_2_O_4_ displayed in Fig. 4A. The histogram of size distribution also displayed in Fig. 4B. The dimension distribution of the particles was determined by measuring100 particles from SEM image using image J software and average grain size was found 70 nm.

### FTIR study of NAFO nanoparticles

**Figure 5:**
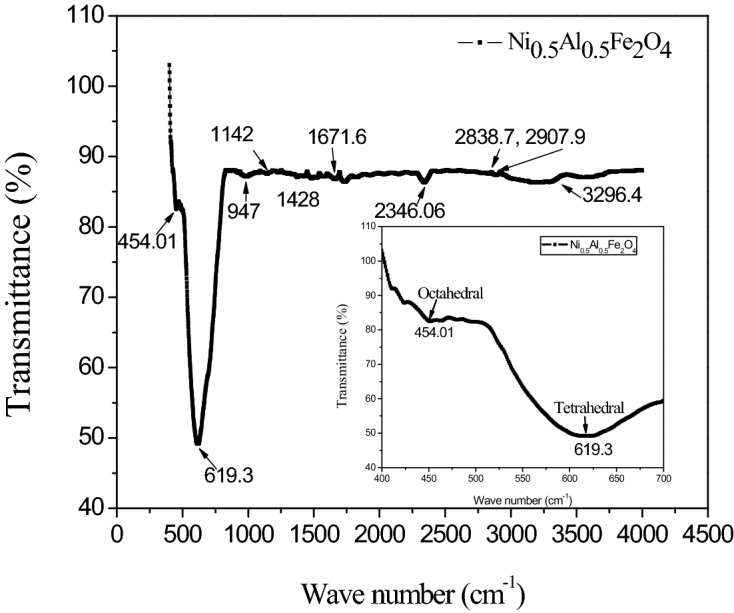
FTIR spectrum of NAFO nanoparticles.

FTIR of the Ni_0.5_Al_0.5_Fe_2_O_4_ nanoparticles were recorded using Perkin-Elmer 1430 IR spectrometer in range 300–4000 cm^−1^ as displayed (Fig. 5). In ferrites as MFe_2_O_4_, two absorption bands occur from interatomic vibrations for the stretching of bonds inside tetrahedral or octahedral oxide ions and metal ions whenever M denotes a divalent metal. The frequency of the band increases due to distribution of Ni, Al and Fe ion over tetrahedral and octahedral sites^40^. Two vibrational modes have appeared at 454.01 and 619.3 cm^−1^. The stretching vibrations of tetrahedrally coordinated (Fe^3+^–O^2−^)) bonds are attributed to the first mode, and the metal oxygen vibrations in the octahedral sites are attributed to the second mode^41^. The absorption band at 947 and 1142 cm^−1^ may be ascribed to the bending vibration of C–H and stretching vibration of C–N. The band at 1428 cm^−1^ was assigned to symmetric vibration (COO_−_) of the carboxylate group bonded to the nanoparticle surface^42^. The bands from 1602–1637 cm^−1^ associated with C = O bonding and 1671 cm^−1^ band is due to the deformation mode of adsorbed water, assigned to the bending vibration^43^. The absorption band of asymmetric and symmetric vibrations of CH_2_ groups appeared at 2838 and 2907.9 cm^−1^ respectively^42^. The band at (3327–3390) cm^−1^ can be attributed to O–H stretching vibrations interacting with H bonds produced from the sample's moisture content^43^. The absorption position in octahedral and tetrahedral complexes of MFe_2_O_4_ crystals differs due to the different distance between Fe3^+^–O^2−^ in the octahedral and tetrahedral sites. The strongest interaction between Fe^3+^ cations and O^2−^ ions at the tetrahedral site resulted in the lowest state of energy due to a difference in electronegativity.

### UV–visible study of NAFO nanoparticles

**Figure 6:**
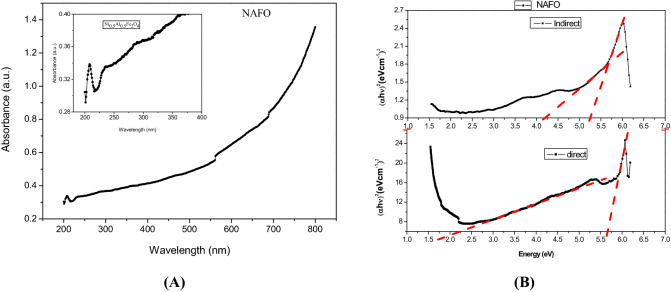
(**A**) UV–vis spectrum (**B**) Tauc’s plot of $(\mathrm{\alpha h\upsilon }$)^2^ (eVcm^−1^)^2^ vs photon energy (eV) of NAFO nanoparticle.

The UV–Visible spectra of NAFO nanoparticles are shown in Fig. 6A. The absorbance of samples was measured in the wavelength range 200–800 nm. The absorption spectrum reveals that the nickel ferrite has low absorbance (~ 206 nm) in visible region and close to infrared region; however, absorbance in UV region is high. The optical band gap can be easily determined from Tauc’s relation^44^ given by Eq. 4

4 $${{\alpha h\upsilon = a}}\left( {{{h\upsilon - E}}_{{\text{g}}} } \right)^{{\text{n}}}$$

where, ‘a’ is a proportionality constant, ‘E_g_’ is the energy band gap of material; hυis energy of photon and value of n depends on types of transition, e.g., *n* = 2 for direct transition and n = 1 /2 for indirect transition. The absorption coefficient α can be expressed as *α* = 2.303 (A_b_/t); where A_b_ is absorbance and t are the thickness of the cuvette or, in powder samples, is the concentration of the solvent. The optical band gap energy of the sample is determined by using Tauc plots, ($\mathrm{\alpha h\upsilon }$)^2^ versus. (hυ), as shown in Fig. 6B. The extrapolation of the straight-line segment gives band gap energy. In this Fig. 6B, more than one linear segment has been seen, but the straight line vertically downward to the X-axis that is chosen to be the value of the band gap. The Tauc plot fit gives two indirect band gap energies at 4.20 eV and 5.25 eV and two direct band gap energies at 2.25 eV and 5.15 eV in the sample which all shown in Fig. 6B, are due to the ideal inverse spinel structure of nickel ferrite. Our result of the optical behavior is analogues to those reported by K. Dileep et.al.^45^. The two band gap energies of sample may be due to the co-existence of high spin and low spin sates in the NiFe_2_O_4_^46^.

### Effect of nanoparticles Ni_0.5_Al_0.5_Fe_2_O_4_ on mycelial growth of pathogen (in vitro)

**Figure 7:**
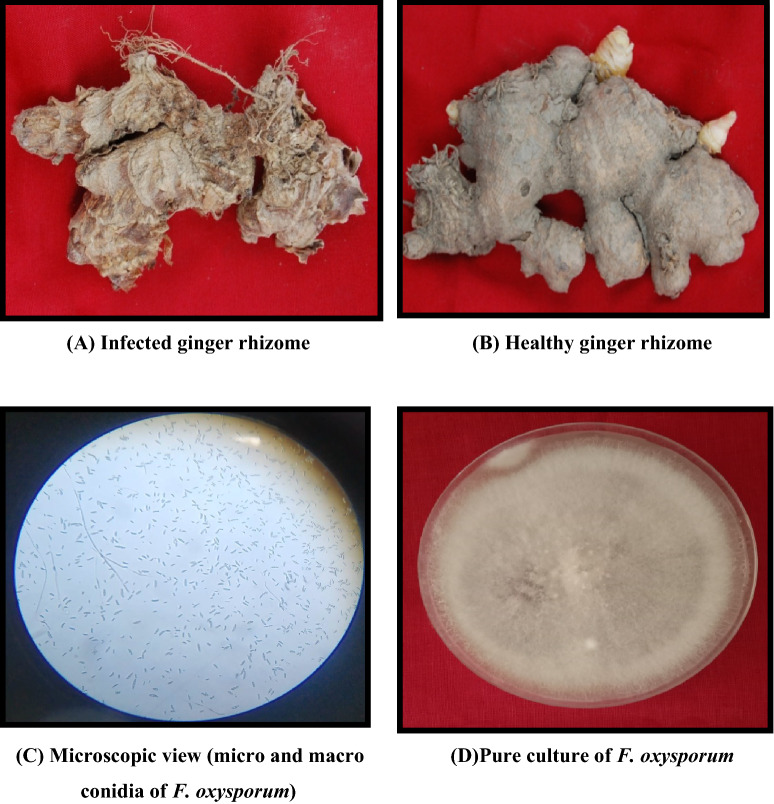
(**A**–**B**) Infected and healthy ginger and (**C**–**D**) Microscopic view and pure culture of dry rot of ginger (*F. oxysporum*).

**Figure 8:**
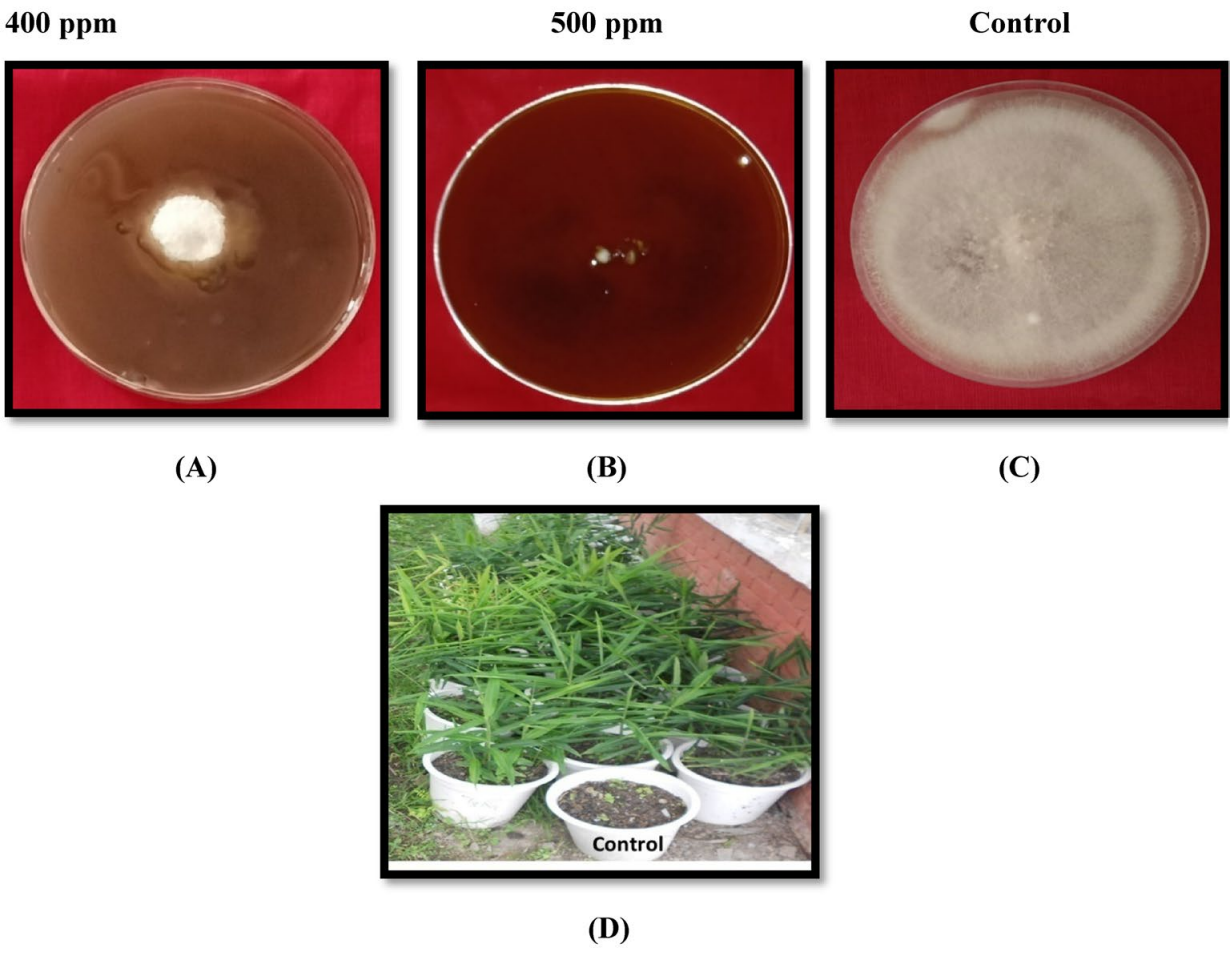
(**A**–**C**) In vitro evaluation and (**D**) In vivo evaluation of antifungal activity of NAFO nanoparticles under pot conditions.

All nanoparticles Ni_0.5_Al_0.5_Fe_2_O_4_ is effective in inhibiting the mycelial growth of the dry rot of the pathogen when compared to untreated control (Fig. 7A, B, C, D). This study indicate that at 500 ppm Fig. 8A (0.5 mg/ml) concentrations of Ni_0.5_Al_0.5_Fe_2_O_4_ under in vitro conditions found to be most effective, results in (91.99%) reduction of the mycelial growth of pathogen followed by 400 ppm Fig. 8B concentration (89.63%); whereas at 100 ppm (0.1 mg/ml) showed minimum (52.77%) reduction of the mycelial growth of *F. oxysporum* in comparison to control as given in Table 3. It was evident from the data that the per cent inhibition of mycelial growth of the dry rot pathogen was increased when we increased the concentration of NiAlFe_2_O_4_ (100 to 500 ppm) nanoparticles (Fig. 8C).The lethal concentration (LC_50_) of nanoparticlesNi_0.5_Al_0.5_Fe_2_O_4_ was found to be 121.62 ppm. The results clearly shows that synthesized nanoparticles having a good antifungal properties against this important plant pathogenic fungus which causes huge damage in various commercially and economically important crops. Similar observation was observed in vivo conditions (Fig. 8D).

**Table 3:** Effect of NAFO nanoparticles against mycelial growth of *F. oxysporum* (in vitro).

	Mycelial growth inhibition (%)							
	100 ppm		200 ppm		300 ppm		400 ppm	500 ppm
*F. oxysporum*	52.77 ± 0.58 (7.33 ± 0.04)		62.53 ± 0.67 (7.97 ± 0.04)		87.63 ± 0.60 (9.41 ± 0.03)		89.63 ± 0.59 (9.52 ± 0.03)	91.99 ± 0.10 (10.04 ± 0.05)
Results are statistically significant								
		Slope (± SE)		LC_50_		Limits 95%	Chi square (χ2)	
*F. oxysporum*		1.25 ± 0.70		121.62 ppm		111.34–156.67	0.85	
C.D		0.105						
SE (m)		0.033						
SE (d)		0.046						
C.V		0.641						

### Determination of minimal fungicidal concentration (MFC) of Ni_0.5_Al_0.5_Fe_2_O_4_ nanoparticles

**Figure 9:**
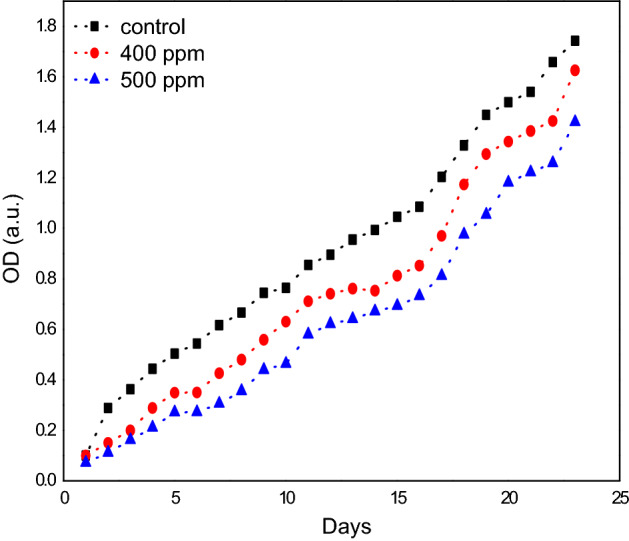
Growth curves of *F. oxysporum* (F.o) at different concentrations of NAFO nanoparticles added at the logarithmic phase. *F. oxysporum* grown without NAFO served as control.

We next wished to determine the minimum concentration at which the NPs were active. We initially desired to investigate the ability of nanoparticles Ni_0.5_Al_0.5_Fe_2_O_4_to affect the growth curves of *F. oxysporum* (F.o.) in different NPs concentrations together with the negative control (*F. oxysporum* grown in suspension without addition of nanoparticles). It was observed that nanoparticles Ni_0.5_Al_0.5_Fe_2_O_4_ inhibited *F. oxysporum* (F.o.) growth, in a concentration-dependent manner. It can be seen (Fig. 9) that viable cell count of the fungus decreased as Ni_0.5_Al_0.5_Fe_2_O_4_ nanoparticles concentration increased. Growing cultures from various time-points of growth were also plated to count viable cells, and the numbers of colony forming cells were consistent with the OD of the growing cultures. As well, nanoparticles Ni_0.5_Al_0.5_Fe_2_O_4_ also inhibited *F. oxysporum* growth when it was added at the logarithmic phase.

### Histidine inhibits the (***F. oxysporum***) antifungal effect caused by Ni_0.5_Al_0.5_Fe_2_O_4_ nanoparticles

**Figure 10:**
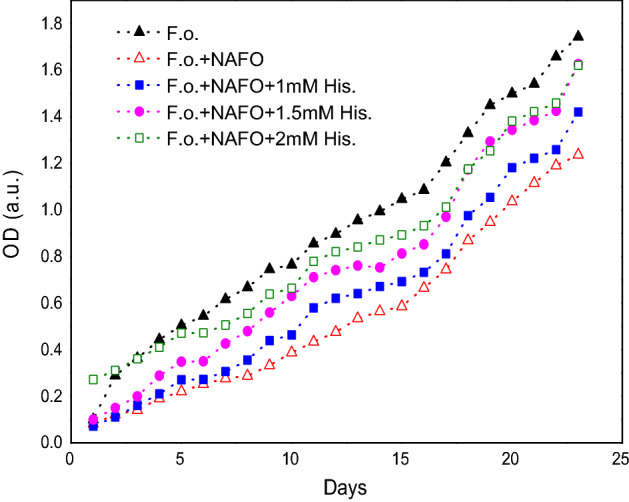
Effect of histidine on the growth curve of *F. oxysporum* with NAFO (500 ppm).

Logarithm growth phase study shows the minimum concentration of nanoparticles (Ni_0.5_Al_0.5_Fe_2_O_4_) act as fungicidal activity. Earlier, studies reported that NPs are having ability of production of ROS (Reactive oxygen species).We examined the role of ROS in nanoparticles (Ni_0.5_Al_0.5_Fe_2_O_4_) represents by reduction in the growth inhibition of *F. oxysporum*. We performed a experiment by adding 1 mM, 1.5 mM and 2 mM histidine in the pathogenic culture of *F. oxysporum* and the effect of histidine at 0.05 mg/ml on nanoparticles (Ni_0.5_Al_0.5_Fe_2_O_4_) mediated toxicity. Figure 10 shows the involvement of histidine on the growth curve of *F. oxysporum* in the presence of nanoparticles (Ni_0.5_Al_0.5_Fe_2_O_4_) (0.05 mg/ml). Histidine (2 mM) almost completely inhibited the antifungal effect of nanoparticles Ni_0.5_Al_0.5_Fe_2_O_4_. Present study is conducted to study by testing whether the ROS produced by the Ni_0.5_Al_0.5_Fe_2_O_4_ nanoparticles. Figure 10 shows a dose dependent effect of histidine on the growth curve of *F. oxysporum* in the presence of Ni_0.5_Al_0.5_Fe_2_O_4_ nanoparticles. Histidine (2.5 mM) almost completely inhibited the fungus and shows antifungal effect of Ni_0.5_Al_0.5_Fe_2_O_4_ nanoparticles. The effect of histidine strongly indicates the role of ROS in the killing of *F. oxysporum*. Study clearly indicates the role and effect of histidine in the production of ROS, how it involve in the cell death by generating singlet oxygen and hydroxyl radical. We speculated in light of these results that the cytotoxic effect of Ni_0.5_Al_0.5_Fe_2_O_4_ nanoparticles on *F.oxysporum* may be mediated through ROS. We can suggest the role of (Ni_0.5_Al_0.5_Fe_2_O_4_) gives a promising results as a fungicidal activity and could be a novel family of future new generation fungicide. Our results suggest that nanomaterials which can induce ROS in water suspensions can also be used for fungal eradication, thus exhibiting another demonstration of the importance of nanoparticles.

### Effect of nanoparticles Ni_0.5_Al_0.5_Fe_2_O_4_ on mycelial growth of pathogen (in vivo)

After successful inhibition of nanoparticles Ni_0.5_Al_0.5_Fe_2_O_4_ under in vitro condition different concentration of nanoparticles were further evaluated for their efficacy in pots in polyhouse conditions. Results indicated that all concentrations of nanoparticles is effective in reduction of the disease incidence of dry rot of ginger (Table 4). Minimum occurrence of disease of dry rot (28.22%) was recorded at (0.5 mg/ml) 500 ppm concentration of Ni_0.5_Al_0.5_Fe_2_O_4_ nanoparticles. Similarly, low disease incidence (39.08%) was recorded when we used (0.4 mg/ml) 400 ppm concentrations of nanoparticles. It is followed by 43.34% at (0.3 mg/ml) 300 ppm concentration of nanoparticles respectively.

**Table 4:** Evaluation of NAFO nanoparticles under pot culture conditions against dry rot of ginger (*F. oxysporum*) (Figures in the parentheses are angular transformed values).

Nanoparticles (NAFO)	Conc (ppm)	Disease incidence (%)
	100	64.30 ± 1.23 (50.89 ± 0.65)
	200	58.02 ± 1.02 (48.60 ± 1.30)
	300	43.34 ± 1.12 (39.88 ± 0.75)
	400	39.08 ± 0.58 (28.67 ± 0.344)
	500	28.22 ± 0.79 (22.67 ± 0.50)
Control		72.34 ± 1.31 (68.56 ± 1.37)
C.D		2.34
SE (m)		0.73
SE (d)		1.03
C.V		2.42
Results are statistically significant		

These results clearly shows that the use of (0.5 mg/ml) 500 ppm of nanoparticles under both in vitro and in vivo conditions resulted in maximum disease reduction followed by treatment with (0.4 mg/ml) 400 ppm of Ni_0.5_Al_0.5_Fe_2_O_4_ nanoparticles.

The current study shows that nanoparticles Ni_0.5_Al_0.5_Fe_2_O_4_ having a strong ability to minimize dry rot of ginger disease incidence, and these nanoparticles could be utilized as an alternative to fungicide for the management of this important disease as it is causes 50–60% losses in storage and field conditions. Antifungal activity of synthesized magnetite nanoparticles NiFe_2_O_4_@Ag and NiFe_2_O_4_@Mo, were assessed against two plant pathogenic fungi *Alternaria solani* and *F. oxysporum* and results indicate that NiFe_2_O_4_@Ag nanoparticle showing highest antifungal activity against these pathogens^47^. The use of nickel nanoparticles as soil drench applications inhibit the Fusarium wilt of tomato and lettuce^48^. Sharma et al.^49^ investigated the antifungal effect against three phytopathogenic fungi by using cobalt and nickel nanoparticles under laboratory and field conditions. Present findings shows that Ni_0.5_Al_0.5_Fe_2_O_4_nanoparticles can be employed as an alternative or effective approach compared to fungicides or pesticides in pest management programmes at the micro and macro levels. Because of their many routes of inhibition, nanoparticles can be used as new antifungal agents and a good alternative to fungicides to prevent the growth of important soil and seed borne disease. Nanoparticles having a good antimicrobial activity even at very low concentrations due to its mode of action (high reactive nature) on target locations.

## Conclusion

Plant diseases are caused by pathogenic bacteria, viruses, fungi, and nematodes, and the contamination/infestation that results causes economic loss. For several years, the potential use of nanoparticles to address the aforementioned requirements has been a source of discussion.

The new generation fungicides is vital to play an important role in emergence of new resistant strains of pathogenic fungi. Research displays the antifungal ability of nanoparticles of against *F. oxysporum*. Moreover, results also indicates that use of nanoparticles have the sufficient ability to reduce the incidence of dry rot of ginger and could be used into practical use in its management. In the present study, under in vivo conditions it was observed that there is no toxicity in ginger plants when we use nanoparticles (Ni_0.5_Al_0.5_Fe_2_O_4_) in 500 ppm concentration. Nanoparticles have both beneficial and toxic effects on plants but the proper doses and right way of application of these nanoparticles helpful in management of important diseases in agriculture. The next study was conducted to examine the effect of nanoparticles on the growth parameters of ginger plant.

## Data Availability

The datasets used or analyzed during the current study were available from the corresponding author on reasonable request.

## References

[1] Adisa IO (2020). Nutritional status of tomato (*Solanum lycopersicum*) fruit grown in Fusarium-infested soil: Impact of cerium oxide nanoparticles. J. Agric. food Chem..

[2] Lipșa FD, Ursu E-L, Ursu C, Ulea E, Cazacu A (2020). Evaluation of the antifungal activity of gold-chitosan and carbon nanoparticles on *Fusarium oxysporum*. Agronomy.

[3] Lucas JA (2009). Plant Pathology and Plant Pathogens.

[4] Kumar MP, Gowda DS, Moudgal R, Kumar NK, Gowda KP, Vishwanath K, Nita M (2013). Impact of fungicides on rice production in India. Fungicides-showcases of Integrated Plant Disease Management from Around the World.

[5] Yin H (2021). Periodic nanostructures: Preparation, properties and applications. Chem. Soc. Rev..

[6] Kora AJ, Mounika J, Jagadeeshwar R (2020). Rice leaf extract synthesized silver nanoparticles: An in vitro fungicidal evaluation against *Rhizoctonia solani*, the causative agent of sheath blight disease in rice. Fungal Biol..

[7] Grover IS, Prajapat RC, Singh S, Pal B (2017). Highly photoactive Au-TiO_2_ nanowires for improved photo-degradation of propiconazole fungicide under UV/sunlight irradiation. Sol. Energy.

[8] Jamdagni P, Rana JS, Khatri P, Nehra K (2018). Comparative account of antifungal activity of green and chemically synthesized zinc oxide nanoparticles in combination with agricultural fungicides. I. J. Nano Dimens..

[9] Consolo VF, Torres-Nicolini A, Alvarez VA (2020). Mycosinthetized Ag, CuO and ZnO nanoparticles from a promising *Trichoderma harzianum* strain and their antifungal potential against important phytopathogens. Sci. Rep..

[10] Antony D, Yadav R, Kalimuthu R (2021). Accumulation of Phyto-mediated nano-CeO_2_ and selenium doped CeO_2_ on *Macrotyloma uniflorum* (horse gram) seed by nano-priming to enhance seedling vigor. Biocatal. Agric. Biotechnol..

[11] Sidhu A, Bala A, Singh H, Ahuja R, Kumar A (2020). Development of MgO-sepoilite nanocomposites against Phytopathogenic fungi of rice (*Oryzae sativa*): A Green Approach. ACS omega..

[12] Eke P, Khanizadeh S (2021). Diagnosis and bioefficacy of endospheric trichoderma strains of selected medicinal plant on pepper root rot and vascular wilt in Cameroon. Archives of Phytopathology and Plant Protection.

[13] Dwivedi S, Saquib Q, Al-Khedhairy AA, Musarrat J, Prodromakis T, Wei B (2016). Understanding the role of nanomaterials in agriculture. Microbial Inoculants in Sustainable Agricultural Productivity.

[14] Gul HT, Saeed S, Khan FZA, Manzoor SA (2014). Potential of nanotechnology in agriculture and crop protection: A. Appl. Sci. Bus. Econ..

[15] Rai M, Kratosova G (2015). Management of phytopathogens by application of green nanobiotechnology: Emerging trends and challenges. Acta Agrar. Debr..

[16] Okey-Onyesolu CF (2021). Nanomaterials as nanofertilizers and nanopesticides: An overview. Chem. Selec..

[17] Zarinkoob A, Esmaeilzadeh Bahabadi S, Rahdar A, Hasanein P, Sharifan H (2021). Ce-Mn ferrite nanocomposite promoted the photosynthesis, fortification of total yield and elongation of wheat (*Triticum*
*aestivum* L.). Environ. Monit. Assess..

[18] Prasad R, Kumar V, Prasad KS (2014). Nanotechnology in sustainable agriculture: Present concerns and future aspects. Afr. J. Biotech..

[19] Rahdar A, Aliahmad M, Samani M, Heidari Majd M, Susan MABH (2019). Synthesis and characterization of highly efficacious Fe-doped ceria nanoparticles for cytotoxic and antifungal activity. Ceram. Int..

[20] Rahdar A, Beyzaei H, Askari F, Kyzas GZ (2020). Gum-based cerium oxide nanoparticles for antimicrobial assay. Appl. Phys. A..

[21] Wu L, He X, Lozano N, Zhang X, Singh PK (2021). ToxA, a significant virulence factor involved in wheat spot blotch disease, exists in the Mexican population of *Bipolaris sorokiniana*. Trop. Plant Pathol..

[22] Ibrahim E, Luo J, Ahmed T, Wu W, Yan C, Li B (2020). Biosynthesis of silver nanoparticles using onion endophytic bacterium and its antifungal activity against rice pathogen *Magnaporthe*
*oryzae*. J. Fungi.

[23] Lamsal K, Kim SW, Jung JH, Kim YS, Kim KS, Lee YS (2011). Application of silver nanoparticles for the control of Colletotrichum species in vitro and pepper anthracnose disease in field. Mycobiology.

[24] Taimoory SM, Rahdar A, Aliahmad M, Sadeghfar F, Hajinezhad MR, Jahantigh M, Shahbazi P, Trant JF (2018). The synthesis and characterization of a magnetite nanoparticle with potent antibacterial activity and low mammalian toxicity. J. Mol. Liq..

[25] Dimkpa CO, McLean JE, Britt DW, Anderson AJ (2013). Antifungal activity of ZnO nanoparticles and their interactive effect with a biocontrol bacterium on growth antagonism of the plant pathogen *Fusarium*
*graminearum*. Biometals.

[26] Rispail N (2014). Quantum dot and superparamagnetic nanoparticle interaction with pathogenic fungi: Internalization and toxicity profile. ACS Appl. Mater. interfaces..

[27] Hassanisaadi M, Barani M, Rahdar A, Heidary M, Thysiadou A, Kyzas GZ (2022). Role of agrochemical-based nanomaterials in plants: Biotic and abiotic stress with germination improvement of seeds. Plant. Growth. Regul..

[28] Hassanisaadi M, Bonjar GHS, Rahdar A, Pandey S, Hosseinipour A, Abdolshahi R (2021). Environmentally safe biosynthesis of gold nanoparticles using plant water extracts. Nanomaterials.

[29] Mohammadzadeh V (2022). Applications of plant-based nanoparticles in nanomedicine: A review. Sustain. Chem. Pharm..

[30] Darr JA, Zhang J, Makwana NM, Weng X (2017). Continuous hydrothermal synthesis of inorganic nanoparticles: Applications and future directions. Chem. Revi..

[31] Sahoo SK, Panigrahi GK, Sahoo A, Pradhan AK, Dalbehera A (2021). Bio-hydrothermal synthesis of ZnO–ZnFe_2_O_4_ nanoparticles using *Psidium*
*guajava* leaf extract: Role in waste water remediation and plant immunity. J. Clean. Prod..

[32] Diba K, Kordbacheh P, Mirhendi S, Rezaie S, Mahmoudi M (2007). Identification of *Aspergillus* species using morphological characteristics. Pakistan J. Med. Sci..

[33] Nahar PB (2008). Effect of repeated in vitro sub-culturing on the virulence of *Metarhizium anisopliae* against *Helicoverpa armigera* (Lepidoptera: Noctuidae). Biocontrol Sci. Tech..

[34] White TJ, Bruns T, Lee S, Taylor J (1990). Amplification and direct sequencing of fungal ribosomal RNA genes for phylogenetics PCR Protoc. Guid. Methods Appl..

[35] Hesham A (2014). New safety and rapid method for extraction of genomic DNA from bacteria and yeast strains suitable for PCR amplifications. J. Pure Appl. Microbiol..

[36] Nene Y, Thapliyal P (1993). Evaluation of Fungicides Fungicides in Plant Disease Control.

[37] Vincent J (1947). Distortion of fungal hyphae in the presence of certain inhibitors. Nature.

[38] Edwards, A., Klug, H. P., Alexander, L. E. in *X-ray diffraction procedures for polycrystalline and amorphous materials: Wiley-Interscience* 2nd edn, (Elsevier, New York, 1975), xxv+, price£ 18.55.

[39] Sharifi S, Yazdani A, Rahimi K (2020). Incremental substitution of Ni with Mn in NiFe_2_O_4_ to largely enhance its supercapacitance properties. Sci. Rep..

[40] Bouhadouza N, Rais A, Kaoua S, Moreau M, Taibi K, Addou A (2015). Structural and vibrational studies of NiAl_x_Fe_2–x_O_4_ ferrites (0≤x≤1). Ceram. Int..

[41] Khairy M, Gouda M (2015). Electrical and optical properties of nickel ferrite/polyaniline nanocomposite. J. Adv. Res..

[42] El-Okr M, Ashery A, Zawrah M, Abou Hammad A (2016). Structural and magnetic analysis on spinel (NiFe_2_O_4_) prepared by sol gel process at different calcinations temperatures. IOSR J. Appl. Phys..

[43] Poh Lin L, Mahmoud Goodarz N, Elias S, Abdul Halim S, Mazaliana Ahmad K (2013). Synthesis and characterization of Ni–Zn ferrite nanoparticles (Ni_0.25_Zn_0.75_Fe_2_O_4_) by thermal treatment method. Adv. Nanoparticles..

[44] Tauc J, Tauc Plenum T (1974). Optical properties of amorphous semiconductors. Amorphous and Liquid Semiconductors.

[45] Dileep K, Polepalli S, Jain N, Buddana SK, Prakasham RS, Murty MSR (2018). Synthesis of novel tetrazole containing hybrid ciprofloxacin and pipemidic acid analogues and preliminary biological evaluation of their antibacterial and antiproliferative activity. Mol. Divers..

[46] Rincón-Granados KL, Vázquez-Olmos AR, Rodríguez-Hernández AP, Vega-Jiménez A, Ruiz F, Garibay-Febles V, Ximénez-Fyvie LA (2021). Facile solid-state synthesis and study in vitro of the antibacterial activity of NiO and NiFe_2_O_4_ nanoparticles. Materialia.

[47] Golkhatmi FM, Bahramian B, Mamarabadi M (2017). Application of surface modified nano ferrite nickel in catalytic reaction (epoxidation of alkenes) and investigation on its antibacterial and antifungal activities. Mater. Sci. Eng. C..

[48] Ahmed AI, Yadav DR, Lee YS (2016). Applications of nickel nanoparticles for control of Fusarium wilt on lettuce and tomato. Int. J. Innov. Res. Sci. Eng. Technol..

[49] Sharma R (2017). Synthesis, antimicrobial activity, structure-activity relationship and cytotoxic studies of a new series of functionalized (Z)-3-(2-oxo-2-substituted ethylidene)-3, 4-dihydro-2H-benzo [b] [1, 4] oxazin-2-ones. Bioorgan. Med. Chem. let..

